# Effects of feeding *Candida utilis*-fermented pea starch on overall, metabolic and intestinal health of dogs and cats

**DOI:** 10.3389/fvets.2025.1542484

**Published:** 2025-06-09

**Authors:** Priscilla Curso-Almeida, Marina Subramaniam, Matheus de Oliveira Costa, Jennifer L. Adolphe, Murray D. Drew, Matthew E. Loewen, Lynn P. Weber

**Affiliations:** ^1^Veterinary Biomedical Sciences, Western College of Veterinary Medicine, University of Saskatchewan, Saskatoon, SK, Canada; ^2^Large Animal Clinical Sciences, Western College of Veterinary Medicine, University of Saskatchewan, Saskatoon, SK, Canada; ^3^Population Health Sciences, Faculty of Veterinary Medicine, Utrecht University, Utrecht, Netherlands; ^4^Animal & Poultry Science, College of Agriculture and Bioresources, University of Saskatchewan, Saskatoon, SK, Canada

**Keywords:** cats, dogs, pea starch, yeast fermentation, intestinal health, metabolic health

## Abstract

Pulse-based pet foods often contain peas or pea starch, which tend to impart a bitter taste. Fermentation increases feed palatability, but also has the potential to improve overall health. Therefore, the current study used the yeast, *Candida utilis,* to ferment pea starch for use in pet food and assessed health effects, focusing on metabolic and intestinal health in dogs and cats. Whole diets had ~30% starch inclusion of either *C. utilis*-fermented pea starch, unfermented pea starch, or a control corn diet fed over a 20-day period to beagle dogs and domestic cats. Complete blood count, biochemistry, adipokines, and triglyceride levels were assessed, along with fecal short chain fatty acids, microbial diversity and abundance to measure intestinal health. It was found that pea-based diets (regardless of fermentation) generally resulted in improved metabolic health by both species, indicated by lower plasma triglycerides, cholesterol, and leptin levels compared to the control corn diet. Additionally, the *C. utilis*-fermented pea starch diet improved dog fecal microbial diversity, while both pea diets increased richness and evenness in the microbial population and a larger *Faecalibacterium* population compared to dogs fed the corn-based diet. In contrast, cat microbiome could not be quantitatively evaluated due to poor fecal quality. Taken together, inclusion of pea starch improves metabolic and intestinal health after 20 days consumption in dogs, while fermentation of pea starch with *C. utilis* may provide additional benefit.

## 1 Introduction

Pulse-based pet foods containing peas or pea starch are known to provide low glycemic starch sources in companion animals that may promote weight control compared to grain-containing diets (1, 2). However, pea starch also imparts a bitter taste that companion animals, particularly cats do not like (2). To ameliorate palatability, fermentation with a variety of microorganisms has been a popular technique used in the pet food industry, as well as other feed industries (3). In fact, a previous study from this research group reported improved palatability in both dogs and cats when the yeast, *Candida utilis,* was used to ferment the pea starch used in test feeds (4). The current study builds off the results of the previous study that used *C. utilis* since it is a robust growing yeast species that does not yield alcohol as a fermentation by-product (5). Yeast fermentation of pea starch also leaves the yeast cell wall in the fermented product, which results in many benefits to humans and animals. This includes polysaccharides such as *β*-glucans that improve human health by lowering cholesterol and exerting anti-inflammatory properties (6). Similarly in dogs, β-D-glucans extracted from oats have had beneficial effects such as reducing total blood cholesterol, as well as other intestinal health benefits (7–9), whereas results with fiber in cats are more equivocal (10–12). Moreover, the yeast cell wall provides a source of fiber and cannot be digested by endogenous enzymes present in the mammalian gastrointestinal tract (13). Therefore, this yeast fiber adds to benefits from other dietary fiber that is already high in peas and further promotes fermentation by intestinal microbes (14). Intestinal microbial fermentation of this fiber or other fermentable fibers yield short-chain fatty acids (SCFAs) such as acetate, butyrate, and propionate, which all benefit intestinal health and the microbial community (13–15).

The distal sections of the mammalian gastrointestinal tract harbors a diverse and abundant population of microbes comprised of bacteria, archaea, fungi, protozoa, and viruses that together represent the gastrointestinal microbiome (13). It is generally considered that the greater diversity and abundance of the gastrointestinal microbial community, the greater the benefits towards host health (16). In fact, intestinal microbial communities are very dynamic and highly susceptible to changes from many factors including gastrointestinal/metabolic diseases (i.e.: obesity), as well as dietary changes in mammals such as dogs and cats (13, 16–18). For instance, it has been hypothesized that obesity may have causal origins in altered gastrointestinal health and microbiome in dogs (19). For example, a high protein-high corn fiber diet in obese dogs that lost weight on high protein/high fiber diets showed improved blood lipids and cholesterol along with alterations in fecal microbiome (20, 21). Thus, while obesity appears linked to metabolic health in dogs (22), effects of specific fiber sources or fractions on blood lipids or other metabolic indicators are unclear. Moreover, the link in cats has yet to be well established. Finally, combined effects of fiber and yeast fermentation product inclusion or fermented ingredient inclusion is unknown in both species.

Taken together, we hypothesized that *C. utilis*-fermented pea starch incorporated into whole diets will improve overall, metabolic, and intestinal health of dogs and cats in comparison to unfermented pea starch or corn diets. A cross-over design was used in beagle dogs and domestic cats, with a feeding period of 20 days per diet, to investigate changes in general health [blood biochemistry, complete blood count (CBC)], metabolic status (weight, plasma leptin and adiponectin and plasma lipids), and intestinal health (fecal SCFA and fecal microbial communities).

## 2 Materials and methods

### 2.1 Animals

Adult mixed sex cats (*N* = 7; neutered/spayed mixed breed; average weight = 4.8 ± 0.5 kg; aged 2–5 years) and adult mixed sex beagle dogs (*N* = 8; neutered/spayed; average weight = 10.8 ± 0.7 kg; aged 3–4 years) were used in this study. This work was approved by the University of Saskatchewan Animal Research Ethics Board (protocol #20130078) following the Canadian Council on Animal Care guidelines. The dogs were obtained from certified scientific breeders (Marshall Farms, North Rose, NY and Liberty Research, Waverly, NY, respectively) and housed at the Western College of Veterinary Medicine at the University of Saskatchewan (Saskatoon, Saskatchewan, Canada). The dogs were group-housed during the day, with free access to an outdoor area, while at night and during feeding periods, the dogs were kept in individual kennels with fresh water provided *ad libitum*. For daily activities, the dogs had playtime and daily walks outdoors with volunteers. In contrast, the cats were allowed to roam freely within the common areas of the animal care facility and had access to a common outdoor courtyard with grass and scratching/climbing structures during the day. At night, the cats were housed individually in kennels with freshwater provided *ad libitum*.

### 2.2 Diets

Three lab-made diets were formulated to have similar proximate composition, differing in the carbohydrate source (Table 1). Pea fiber was added to all diets including the corn diet, but both the pea fiber and soy protein were used to balance diets to be isonitrogenous and isocaloric. Since the same diets were fed to both dogs and cats, the diets were formulated to meet the higher minimum protein, taurine, and other nutritional needs of cats (71). While the lab-made diets exceeded nutritional requirements for dogs, they resembled many of the premium dog diets on the commercial market (23). Test diets were formulated with unfermented wet-processed purified pea starch (Parrheim Foods, Saskatoon, SK) or this same starch after fermentation with *Candida utilis* according to methods described previously (4). The cornstarch was purchased from a local grocery store (Whole Sale Club, Saskatoon, SK Canada). Proximate analyses for macronutrient composition, energy (as metabolizable energy or ME), and acid insoluble ash (Celite incorporated at 1%) for each lab-made test diet were performed at a commercial testing lab (Central Testing, Winnipeg, MB; Table 1) according to the AOAC standard methods [see Table 1 for specific methods used (24)]. This included measurements of dry matter (oven-dried samples), non-fiber carbohydrates, crude protein using the Kjeldahl method, and fat analysis through acid-hydrolysis solvent extraction, which were analyzed in diets and feces according to the AOAC standards, and previously published with the same cohort of animals in this group (4, 24). The non-digestible Celite marker was also used to determine total tract apparent digestibility and in proximate analyses of diet and feces.

**Table 1:** Proximate analyses of test diets with listed ingredients in decreasing order of inclusion (% dry matter, as fed) formulated for the feeding trial.

Nutrient (%DM)	Control (corn) diet	Unfermented pea diet	Fermented pea diet
Protein^a^	38.4	37.4	37.9
Fat^b^	14.2	15.7	12.7
Non-fiber carbohydrates^c^	32.0	30.9	32.7
Crude Fiber^d^	3.2	4.8	4.9
ME (Kcal/Kg)^e^	3,760	3,748	3,550

Ingredients	Control (corn) diet (%)	Unfermented pea diet (%)	Fermented pea diet (%)
Carbohydrate source	30.08	31.92	31.92
Chicken meal	35.78	25.46	25.46
Soy protein concentrate	9.31	15	15
Chicken fat with Dadex T	7.75	9.76	9.76
Pea fiber (PetFine^®^)	6.88	4.96	4.96
Fish meal, mixed	5.0	5.0	5.0
Liquid palatant (AFB LC647 LQDGST)	2.0	2.0	2.0
Fish oil	2.0	2.0	2.0
Celite	1.0	1.0	1.0
Potassium chloride	0.9	0.88	0.88
Sodium chloride	0.3	0.5	0.5
Dry palatant (AFB F24047 Dry cat)	0.5	0.5	0.5
Calcium carbonate	–	0.41	0.41
Choline chloride	0.1	0.2	0.2
Methionine/DL	0.1	0.1	0.1
Mineral premix, dog cat	0.1	0.1	0.1
Vitamin premix, cat	0.1	0.1	0.1
Taurine	0.1	0.1	0.1
Dicalcium phosphate	–	0.01	0.01

DM, dry matter; ME, metabolizable energy.

a Determined by Central Testing Laboratory Ltd. (Winnipeg, MB, Canada) using method AOAC 990.03 using a conversion factor of 6.25.

b Determined by Central Testing Laboratory Ltd. (Winnipeg, MB, Canada) using method AOAC A 5–04.

c Determined by Central Testing Laboratory Ltd (Winnipeg, MB, Canada) using equation Non-Fiber Carbohydrates (DM): 100 – [(% Dry Matter/100) + % Protein + %Fat + %Ash + %Crude Fiber].

d Determined by Central Testing Laboratory Ltd. (Winnipeg, MB, Canada) using method by Ankom Technologies (2017) based on AOCS Ba 6a-05.

e Determined by Central Testing Laboratory Ltd. (Winnipeg, MB, Canada) using the ME equation for swine: (kcal/kg) = 4,151-(122*Ash) + (23*Crude Protein) + (38*Fat)-(64*Crude fiber)*(1.003-(0.0021* Crude Protein)) by Central Testing Laboratory Ltd. (Winnipeg, MB, Canada).

Diets were formulated using Concept 5 software (Creative Formulation Concepts, LLC, MD, United States) according to Association of American Feed Control Officials (AAFCO) nutrient requirements for cats (71). The diets were extruded at the Food Science and Technology Centre (FSTC) in Brooks, Alberta, Canada, using a Coperion ZSK57 extruder (Werner & Pfleiderer, Ramsey, NJ, United States). The extruder consisted of 8 barrels on which the temperature was independently controlled by Mokon oil heaters (HC5X12-Q1, Mokon, Buffalo, NY, United States). At the end of the process, the pellets were coated with a mixture of chicken fat and fish oil with a fluidizing paddle blender (FPB-20, American Process Systems, Gurnee, IL, United States). Finally, the diets were coated first with a commercial liquid palatant (AFB LC647 LQDGST, AFB St. Charles, MO, United States), followed by a dry palatant (AFB F24047 Dry Cat) at the Canadian Feed Research Centre in North Battleford, SK.

### 2.3 Feeding, body condition scores, and body weight

Both species were fed species-specific balanced commercial dry pet food (husbandry diet) containing chicken and either barley or corn as the top ingredients (Hill’s Science Diet, Hill’s Pet Nutrition, Inc. Topeka, United States), except for the digestibility and feeding trial periods where test diets were fed instead. The calories per day needed to maintain optimal body condition score for each individual animal was determined in the pre-trial period of at least 2 months on the commercial husbandry diet and conformed to the National Research Council guidelines (25). Both species were maintained at a standard body condition score on a 9-point scale, where 5/9 were ideal for cats and 4–5/9 were ideal for dogs (4, 26, 27).

### 2.4 Digestibility trial and total tract apparent digestibility

The digestibility trial was conducted prior to the feeding trial (*n* = 7 cats, *n* = 8 dogs). During the digestibility and feeding trials, each animal was fed twice daily with total daily portions of test diets with equivalent metabolizable energy (ME) to that calculated to maintain optimal body condition during the pre-trial period. During both trials, the test diets were formulated with ~30% inclusion of either corn (control), unfermented pea starch, or fermented pea starch (4); (see Table 1 for formulation). The level of starch inclusion was chosen to be 30%, since higher levels of pulse starches have been known to cause gastrointestinal issues (28). Additionally, 30% pea starch inclusion in previous studies have shown lower postprandial insulin responses after a glucose challenge, as well as increased insulin sensitivity and weight control in dogs (1, 2, 4). Finally, pet foods generally contain 30% starch inclusion in extruded kibble diets (29, 30). The digestibility trial lasted 7 days for each diet and the same diet was fed to all dogs for a given 7-day period in the following order: corn, unfermented pea starch, and fermented pea starch diets with no washout periods in-between. For the digestibility trial, dogs were kept in individual kennels on days 6–7 of feeding and feces were collected. For dogs, feces were collected immediately upon defecation from the kennel floor, starting lights on at 7 am before feeding in the morning (defecation was greatest immediately after each meal). Dog feces was continued to be collected the entire day until lights out at 7 pm or when 5 g of feces had been collected, whichever came first. All fecal samples collected for digestibility for both species were a minimum of 3 g for each individual. For cat digestibility fecal collections, cats were kept in individual kennels with non-absorbent litter in their litter box within the kennel starting before the evening meal on Day 6. Cat feces was collected from the individual litter box the next day and cats kept in individual kennel until sufficient feces collected. Once collected for both species, feces from the sampling period were pooled for a given individual, mixed to ensure homogeneity and then frozen at −20°C until proximate analyses.

For digestibility, fecal samples were dried in a forced-air oven at 65°C for 72 h and then ground (23). Apparent digestibility was determined as total tract apparent digestibility according to the following equation where the indicator used was 1% Celite as included in each test diet (see Table 1 for diet formulations) and was measured as acid-insoluble ash (31):


$$\text{Apparent Digestibility}\;(\%)=\{\begin{array}{l} 1-\frac{\left(\%\text{Nutrient in Feces}\right)}{\left(\%\text{Nutrient in Diet}\right)}\\ \times \frac{\left(\%\text{Indicator in Diet}\right)}{\left(\%\text{Indicator in Feces}\right)} \end{array}\}\times 100$$

### 2.5 Feeding trial

The feeding trial (*n* = 7 cats, *n* = 8 dogs) was conducted after at least a month pre-trial period that followed the digestibility trial. It consisted of three 20-day feeding periods and used the same test diets from the digestibility trial, with 15-day wash-in/−out period on the commercial husbandry diet between test periods. The sequence of feeding and washout periods are outlined in Table 2. The test diets with corn and ~ 30% inclusion of fermented and unfermented peas were provided twice daily during periods 1, 2, or 3 in a randomized, cross-over manner so that all dogs/cats had received each test diet. The main personnel (Curso-Almeida) collecting data and feeding was blinded to the diet being fed. Each day, the remaining food was saved, weighed and subtracted from the total offered to determine individual daily feed intake. Finally, at the end of the feeding period for each test diet, individual body weights were recorded, and body condition scores (BCS) were assessed by the same individual using a 9-point scale (26, 27).

**Table 2:** Sequence and duration of the three test feeding periods plus wash in/out periods.

Feeding trial periods	Diets	Duration (days)
Pre-Trial Phase	Commercial	62
Wash-in Phase	Commercial	15
Test Period 1	Test Diet 1	20
Wash-out Phase	Commercial	15
Test Period 2	Test Diet 2	20
Wash-out Phase	Commercial	15
Test Period 3	Test Diet 3	20

Periods are listed in sequential order from first (top) to last (bottom).

### 2.6 Fecal collection for short chain fatty acids from feeding trial

In the last 3 days of each feeding trial period, approximately 1 g fresh feces was collected rectally to measure short-chain fatty acid (SCFA) content. To ensure integrity of SCFA content, rectal palpation was used to collect fecal samples from dogs, and for cats, the process involved careful observation for defecation followed by immediate fecal sample collection to prevent the sample from touching the floor or litter box. Additionally, to avoid loss of volatile compounds, feces were immediately placed after defecation in a sealed tube containing a mixture of water and 25% m-phosphoric acid (4:1). After vortexing, samples were allowed to precipitate for 30 min, centrifuged at 20,000 x g for 20 min at 4°C, supernatant collected and stored at −80°C until SCFA concentrations were determined by gas chromatography (32).

### 2.7 Blood collection, adipokines and triglycerides

After 20-day feedings of each diet, blood collected from the cephalic vein of fasted dogs and cats was sent to Prairie Diagnostic Services (University of Saskatchewan, SK, Canada) for total blood cell count and biochemistry profile. Additional blood samples were placed in EDTA tubes and centrifuged at 5,000 x g for 10 min. The plasma was collected and frozen at −80°C, until assays were run to measure adipokine levels and total triglycerides. Dog adiponectin was determined using a species-specific ELISA kit (Circulex dog adiponectin ELISA kit, MBL international, Massachusetts, United States). Cat adiponectin was determined using a human ELISA kit validated for cats (33) (Adiponectin Sandwich High Sensitivity, Biovendor, Brno, Czech Republic). Dog and cat leptin plasma concentrations were determined using a canine ELISA assay (Canine Leptin ELISA kit, Millipore Sigma, Oakville, ON, Canada). Plasma total triglycerides of dogs and cats were determined using a glycerol kinase-based method for the colorimetric determination of triglycerides (34) (Sigma-Aldrich, Oakville, ON, Canada).

### 2.8 Microbiome analyses

Sterile dry transport swabs were used to collect rectal fecal samples from dogs and cats. Samples were kept at −80°C until extraction and sequencing. DNA content from swabs was extracted using the PowerSoil Max DNA Kit (Mo Bio, Carlsbad, California, United States) following the manufacturer’s instructions. Reagent blanks were included in the extraction process and remained free from DNA contamination during this process. Total DNA extracted from samples was measured prior to library preparation using both NanoDrop spectrophotometer (ND-1,000, NanoDrop Technology, Wilmington, Delaware, United States) and PicoGreen dsDNA assay (Life Technologies, Carlsbad, CA, United States). Sequencing was performed using a protocol previously described (35). Briefly, amplification of the 16S rRNA gene V3-V4 hypervariable region used a KAPA HiFidelity Hot Start Polymerase (Kapa Biosystems Inc., Wilmington, MA, Unites States) and Nextera primers (Meta_V3_F_Nextera: 5′-CCTACGGGAGGCAGCAG-3′, Meta_V4_806_R: 5′-GACTACHVGGGTWTCTAAT-3′, Integrated DNA Technologies, Coralville, IA, USA). The first round of amplification used the following cycling parameters: one cycle of 95°C for 5 min, followed by 20 cycles of 98°C for 20 s, 55°C for 15 s, and 72°C for 1 min. The second round used indexing primers (F: 5′- AATGATACGGCGACCACCGAGATCTACAC[i5]TCGTCGGCAGCGTC-3′, R: 5′- CAAGCAGAAGACGGCATACGAGAT[i7]GTCTCGTGGGCTCGG-3′) and the following cycling conditions: 1 cycle at 95°C for 5 min, followed by 10 cycles of 98°C for 20 s, 55°C for 15 s, and 72°C for 1 min. We chose to verify whether amplification was successful by performing electrophoresis of amplicons on 1% agarose. Pooled, size-selected samples were denatured with NaOH, diluted to 8 pM in Illumina’s HT1 buffer, spiked with 15% PhiX, and heat denatured at 96°C for 2 min immediately prior to loading. The MiSeq 600 (2 × 300 base pairs, bp) cycle v3 kit (Illumina, San Diego, CA, United States) system was used to sequence DNA libraries. Data were analyzed using QIIME2 (36).

### 2.9 Data analyses and statistics

Sequencing data were demultiplexed and sorted by barcodes to generate fastq files for each individual sample. Raw amplicon reads were filtered by trimming the first 13 bp, and truncating forward and reverse reads at 250 bp using the Dada2 plug-in (35). Filtered reads were dereplicated and denoised using parameters estimated for this dataset. Forward and reverse sequences were merged using a minimum overlap of 20 bp and 0 mismatches were allowed with Dada2. Representative sequences were classified against the SILVA SSU Ref NR dataset v.128 at 98% sequence similarity using a classifier algorithm trained for this work’s dataset (37). Next, contaminant sequences (non-bacterial, mitochondrial, or chloroplast DNA) and sequences not classified beyond the kingdom level were removed from the dataset using the quality control plug-in.

Statistical analyses were performed using IBM SPSS version 24 (IBM Corp., Armonk, NY). Data was tested for normality of distribution using a Kolmogorov–Smirnov test and homogeneity of variance using a Levene’s test. Most data met parametric assumptions and a paired t-test or repeated measures one-way ANOVA using a generalized linear model followed by Fischer’s LSD posteriori tests were used, as appropriate. For non-parametric data, Friedman’s ANOVA using a rank-based model was performed (BCS). Differences were considered significant at P < 0.05. DNA sequencing data was analyzed separately using Phyloseq, as previously described (38). Alpha diversity was determined using Friedman’s test, followed by Dunn’s multiple comparison *post hoc* tests. Beta diversity analyses were conducted using PERMDISP and PERMANOVA. Differences in taxa abundance were determined using Friedman’s test, followed by Dunn’s multiple comparison tests. False discovery ratio (FDR > 2-fold) correction was applied to the calculated *p* values. Differences were considered significant at p and q < 0.05.

## 3 Results

### 3.1 Total tract apparent digestibility

Dogs fed the fermented pea diet show significantly higher protein, starch and energy total tract apparent digestibility (TTAD) compared to the unfermented pea diet, but no change in fat digestibility (*p* < 0.05, Paired-sample *t*-test; Table 3). In contrast, in cats, only starch digestibility was significantly higher with the fermented pea diet in comparison to the unfermented pea diet (*p* < 0.05, Paired-sample *t*-test; Table 3).

**Table 3:** Apparent digestibility coefficient for dogs and cats fed the unfermented or fermented pea diets.

Species	Diet (%DM)	Protein (% DM)	Fat (% DM)	Starch (% DM)	DE (kcal/kg)
Dog	UPD	84 ± 0.8ᵃ	98 ± 0.1	81 ± 1.0ᵃ	83 ± 0.5ᵃ
	FPD	86 ± 0.4ᵇ	98 ± 0.1	86 ± 0.2ᵇ	84 ± 3.0ᵇ
Cat	UPD	83 ± 0.7	95 ± 1.0	85 ± 1.0ᵃ	82 ± 1.0
	FPD	85 ± 0.4	97 ± 0.2	89 ± 0.4ᵇ	84 ± 0.3

Data is mean ± SEM. Dogs *n* = 8, cats *n* = 7. Paired-sample *T*-test. Values in a column with superscripts without a common letter are significantly different (*p* < 0.05). UPD, unfermented pea diet; FPD, fermented pea diet; DE, digestible energy; DM, dry matter.

### 3.2 Feed intake, body weight, and body condition score

**Figure 1:**
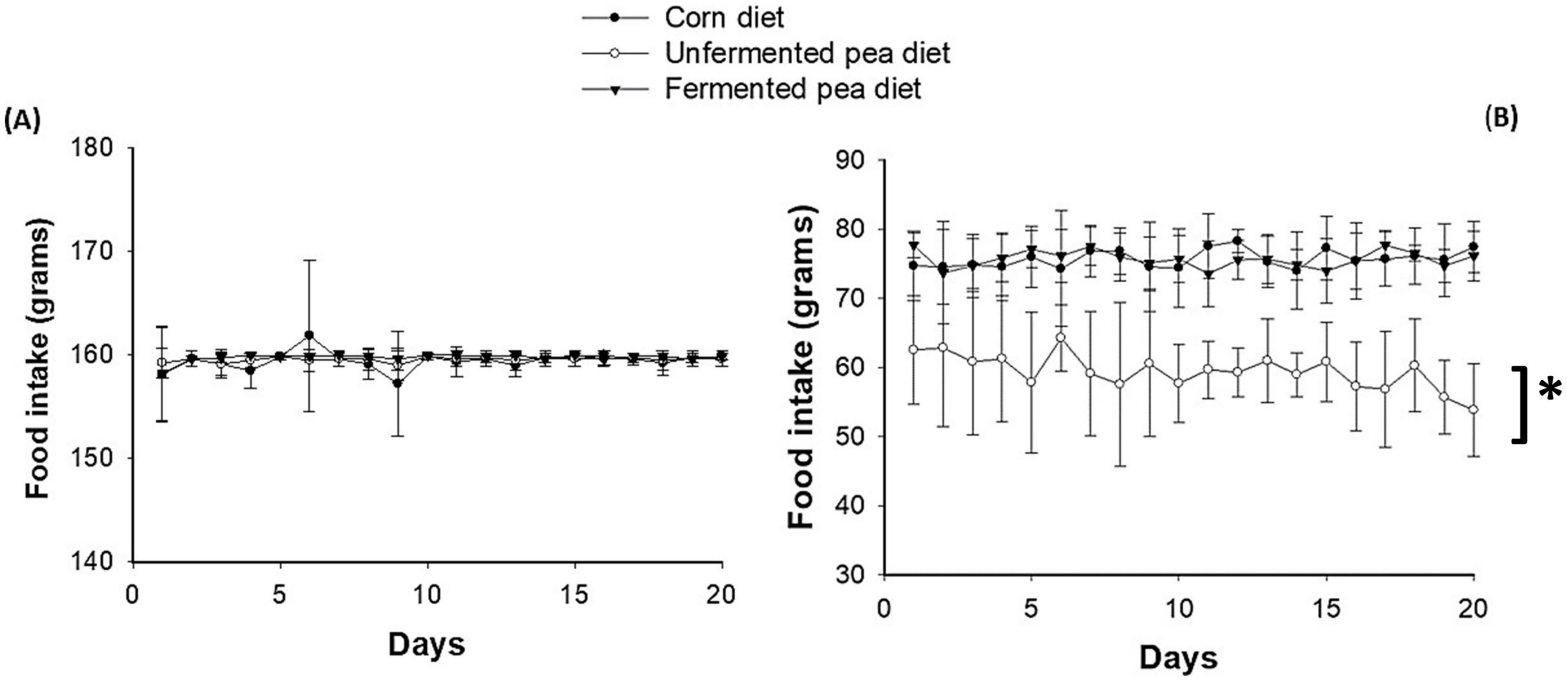
Daily food intake. Values are mean ± SEM for **(A)** dogs (*n* = 8) and **(B)** cats (*n* = 7). One-way repeated measures ANOVA followed by Tukeys test. An asterisk represents significance for the cats between unfermented pea diet compared to fermented pea and corn diets (*p* < 0.05).

In dogs, no difference in feed intake was found regardless of the diet fed (Figure 1). All food was pre-portioned and dogs readily ate all food regardless of diet within 5 min. No significant differences in body weight and body condition score were found among dogs after feeding either pea-based or the control corn diets (Table 4). Cats also received individually defined food portions each day. However, unlike the dogs, cats showed food refusals and significantly reduced feed intake when fed the unfermented pea diet compared to either the corn or the fermented pea diets (*p* < 0.05, Fischer’s LSD test after one-way repeated measures ANOVA; Figure 1). As a result, statistically significant weight loss was observed when cats were fed the unfermented pea diet compared to the corn, but not the fermented pea diet (*p* < 0.05, Table 4). Despite the differences observed in body weight, no significant difference was found in cat body condition score after feeding the different diets (Table 4).

**Table 4:** Body weight and body condition score of dogs and cats after fed test diets for 20 days.

Species	Corn diet	UPD	FPD
Body weight			
Dogs	10.8 ± 0.2	10.6 ± 0.2	10.7 ± 0.2
Cats	4.8 ± 0.2ᵃ	4.6 ± 0.3ᵇ	4.7 ± 0.2ᵃᵇ
Body condition score			
Dogs	6.1 ± 0.2	5.8 ± 0.2	5.6 ± 0.2
Cats	5.5 ± 0.2	5.0 ± 0.3	5.2 ± 0.2

Data is mean ± SEM. Dogs *n* = 8, cats *n* = 7. Body Weight: One-way repeated measures ANOVA. Values in a row with superscripts without a common letter are significantly different (*p* < 0.05). Body Condition score: Friedman’s ANOVA test. No significant differences. UPD, unfermented pea diet; FPD, fermented pea diet.

### 3.3 Blood biochemistry

For the dogs, significant changes in blood biochemistry were observed, but all blood values after feeding each diet remained within the reference range (Supplementary Table S1). Specifically, the dogs showed a significant decrease in serum amylase concentration with the fermented pea diet (*p* < 0.05, Supplementary Table S1). Additionally, in comparison with the corn and fermented pea diets, dog plasma cholesterol levels were significantly lower when they received the unfermented pea diet (*p* < 0.05, Supplementary Table S1). Overall, no differences in fasting blood glucose concentration, blood urea and creatinine concentrations, as well as blood bilirubin concentrations were observed in dogs regardless of diet (Supplementary Table S1).

In cats, all the values except for cholesterol were within the reference range (Supplementary Table S2). In fact, the values for cholesterol in cats were already outside the higher end of the reference range before starting this feeding trial. Cholesterol levels revealed significant differences, with the lowest values attributed to the unfermented pea diet, followed by the fermented pea diet, and then the corn diet (*p* < 0.05, Supplementary Table S2). Amylase levels were not part of the standard chemistry panel for cats measured by the clinical chemistry service (Prairie Diagnostics, Saskatoon, SK Canada). Of interest, fasting blood glucose concentrations were significantly higher in cats fed the fermented pea diet, followed by the unfermented and corn-based diets (*p* < 0.05, Supplementary Table S2). Additionally, the blood urea was significantly increased for both the corn and fermented pea diets in comparison to the unfermented pea diet (*p* < 0.05, Supplementary Table S2). However, creatinine and bilirubin concentrations were significantly decreased after consuming only the corn diet compared to the other diets (*p* < 0.05, Supplementary Table S2).

### 3.4 Total triglycerides

In comparison to the corn diet, total plasma triglycerides were significantly reduced for both dogs and cats with both pea-based diets (*p* < 0.05, Table 5).

**Table 5:** Total triglycerides of dogs and cats after fed test diets for 20 days.

Triglycerides (mg/dL)	Reference range*	Corn diet	UPD	FPD
Dogs	29–291	481.5 ± 50ᵃ	225.3 ± 30ᵇ	186.6 ± 19ᵇ
Cats	25–160	340.0 ± 66ᵃ	167.5 ± 29ᵇ	161.2 ± 15ᵇ

*(72). Values are mean ± SEM. Dogs *n* = 8, cats *n* = 7 for cats. One-way repeated measures ANOVA with post-hoc LSD. Values in a row with superscripts without a common letter are significantly different (*p* < 0.05). UPD, unfermented pea diet; FPD, fermented pea diet.

### 3.5 White blood cells, neutrophil-lymphocyte ratio (NLR) and platelet-lymphocyte ratio (PLR)

Total white blood cell count (WBC) was significantly lower in dogs fed both pea-based diets in comparison to the corn (*p* < 0.05, Supplementary Table S1). Total counts of blood neutrophils and lymphocytes were higher in dogs fed the corn diet compared to both pea-based diets, without any difference between fermented and unfermented pea diets (Table 6). In contrast, no significant differences were observed in total blood platelets count and NLR, regardless of diet. However, dogs fed both pea-based diets had similar PLR levels, which were both significantly higher than the corn diet (*p* < 0.05, One-way repeated measures ANOVA; Table 6).

**Table 6:** Dog and cat neutrophil, lymphocyte and platelet counts plus NLR (neutrophil:lymphocyte ratio) and PLR (platelets:lymphocyte ratio) after fed test diets for 20 days.

Blood Parameter	Corn diet	UPD	FPD
Dog			
Neutrophils (x10⁹/L)	3,705 ± 234ᵃ	2,882 ± 277ᵇ	3,009 ± 244ᵇ
Lymphocytes (x10⁹/L)	1909 ± 216ᵃ	1,390 ± 106ᵇ	1,358 ± 112ᵇ
Platelets (x10⁹/L)	224 ± 19	228 ± 26	225 ± 36
NLR	2.0 ± 0.2	2.2 ± 0.3	2.3 ± 0.2
PLR	0.12 ± 0.1ᵃ	0.17 ± 0.2ᵇ	0.17 ± 0.1ᵇ
Cat			
Neutrophils (x10⁹/L)	5,177 ± 778	4,960 ± 472	5,321 ± 532
Lymphocytes (x10⁹/L)	4,584 ± 790	4,918 ± 748	5,504 ± 1,044
Platelets (x10⁹/L)	224 ± 19	228 ± 26	225 ± 36
NLR	1.5 ± 0.5	1.2 ± 0.3	1.1 ± 0.2
PLR	0.06 ± 0.07ª	0.06 ± 0.10ª	0.04 ± 0.09ᵇ

Data is mean ± SEM, *n* = 8 dogs; *n* = 7 cats. One-way repeated measures ANOVA. Values in a row with superscripts without a common letter are significantly different (*p* < 0.05). UPD, unfermented pea diet; FPD, fermented pea diet; baseline, corn diet, NLR, neutrophil/lymphocyte ratio; PLR; platelet/ lymphocyte ratio.

No difference in WBC count was observed in cats regardless of diet. In cats, no significant differences were observed in the total count of blood neutrophils, lymphocytes, or NLR among diets (Table 6). However, the PLR value was significantly lower when cats consumed the fermented pea diet compared to either corn or the unfermented pea diet (*p* < 0.05, One-way repeated measures ANOVA; Table 6).

### 3.6 Plasma adipokines

**Figure 2:**
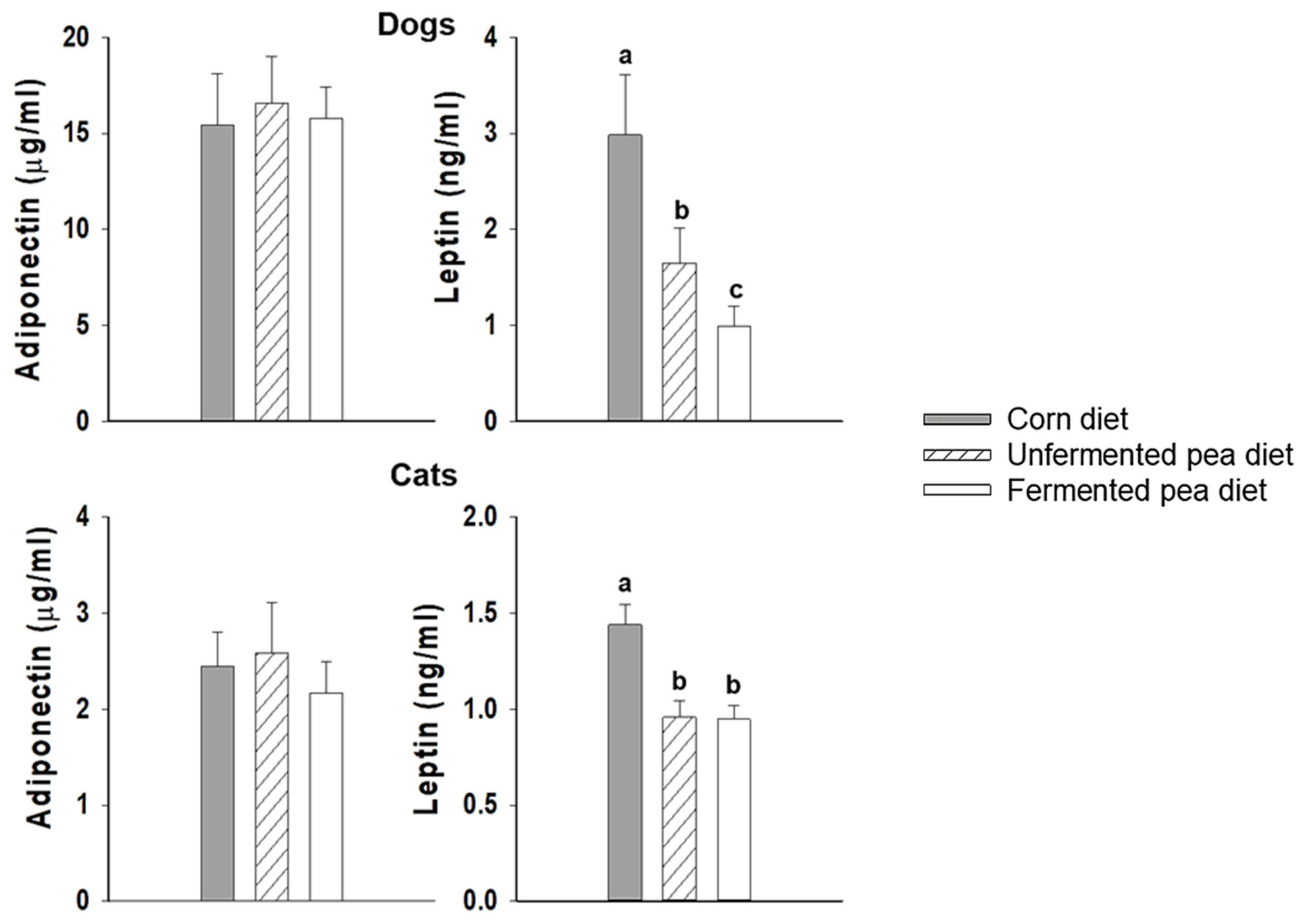
Adipokine levels of dogs and cats after feeding test diet for 20 days. Values are mean ± SEM for dogs (*n* = 8) and cats (*n* = 7). One-way repeated measures ANOVA with post-hoc LSD. Bars with superscripts without a common letter are significantly different (*p* < 0.05).

No significant changes in adiponectin concentration were observed in dogs or cats, regardless of diet (Figure 2). For the leptin levels, not only did dog leptin concentration significantly decrease with the unfermented pea diet, but it was further reduced with the fermented pea diet (*p* < 0.05, Figure 2). In contrast, cats showed similarly decreased leptin concentrations for both the fermented and unfermented pea-based diets compared to the corn diet (*p* < 0.05, Figure 2).

### 3.7 Fecal short-chain fatty acids

**Figure 3:**
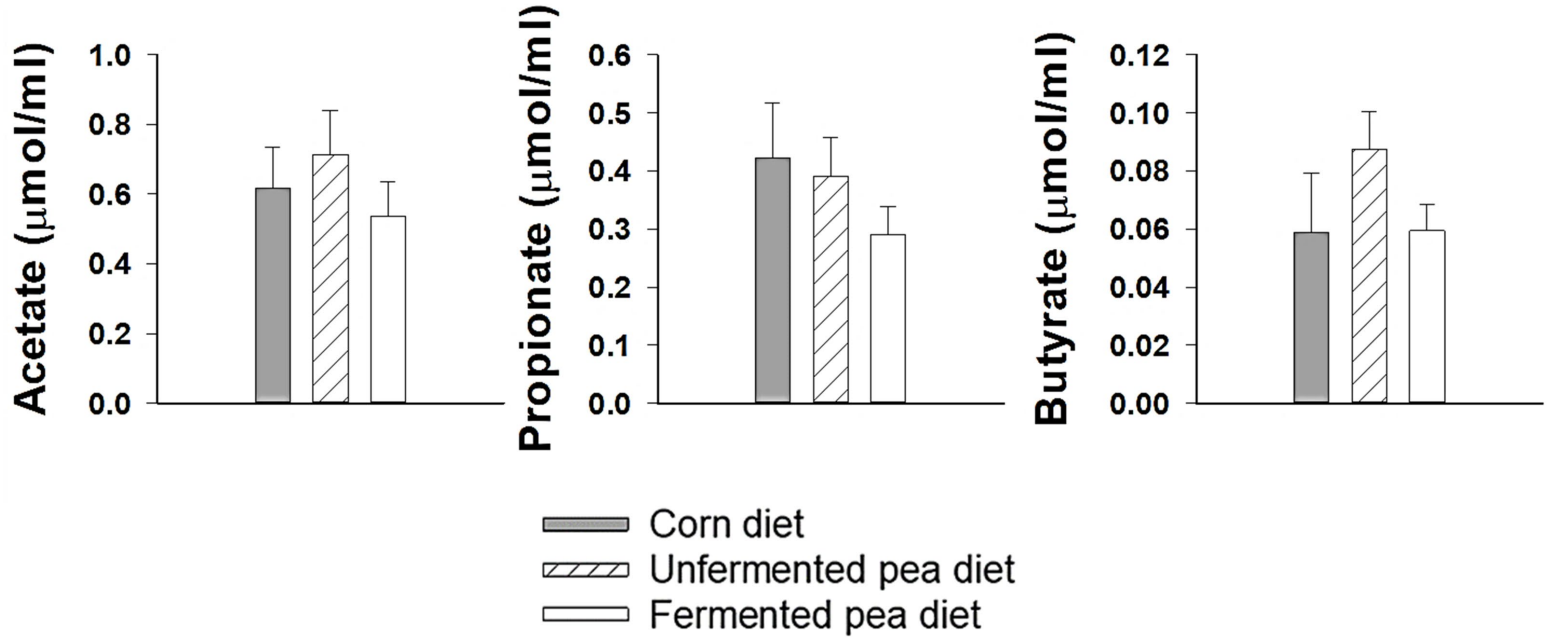
Fecal short-chain fatty acid concentration of dogs after being fed test diets for 20 days. Values are mean ± SEM (*n* = 8). No significant differences detected using one-way repeated measures ANOVA.

**Figure 4:**
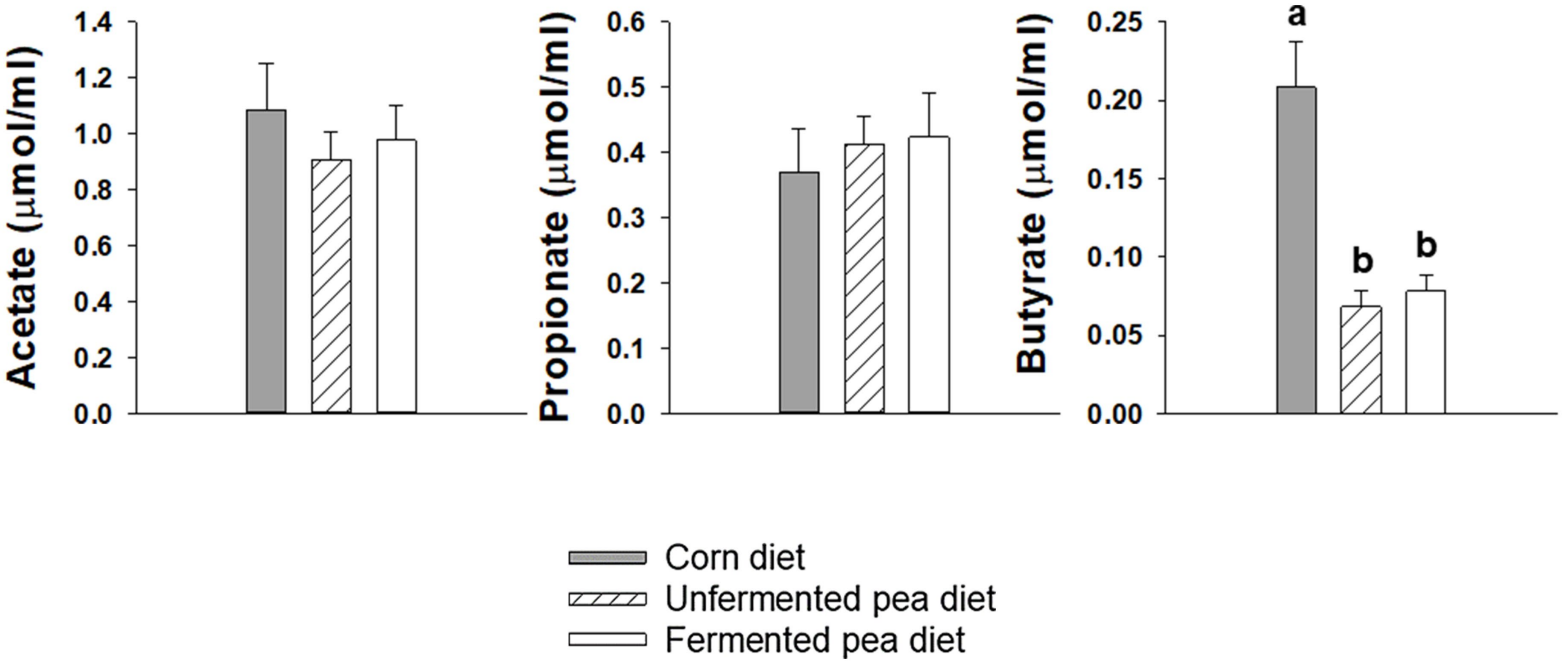
Fecal short-chain fatty acid concentration of cats after being fed test diets for 20 days. Values are mean ± SEM (*n* = 7). One-way repeated measures ANOVA with post-hoc LSD. Bars with superscripts without a common letter are significantly different (*p* < 0.05).

In dogs, no significant differences were found in fecal SCFAs: acetate, propionate, or butyrate among diets (Figure 3). Similarly, fecal acetate and propionate levels did not significantly differ between any of the diets for cats (Figure 4). However, fecal butyrate concentration was significantly lower for both pea-based diets compared to the corn diet in cats (*p* < 0.05, Figure 4).

### 3.8 Fecal microbiome

**Figure 5:**
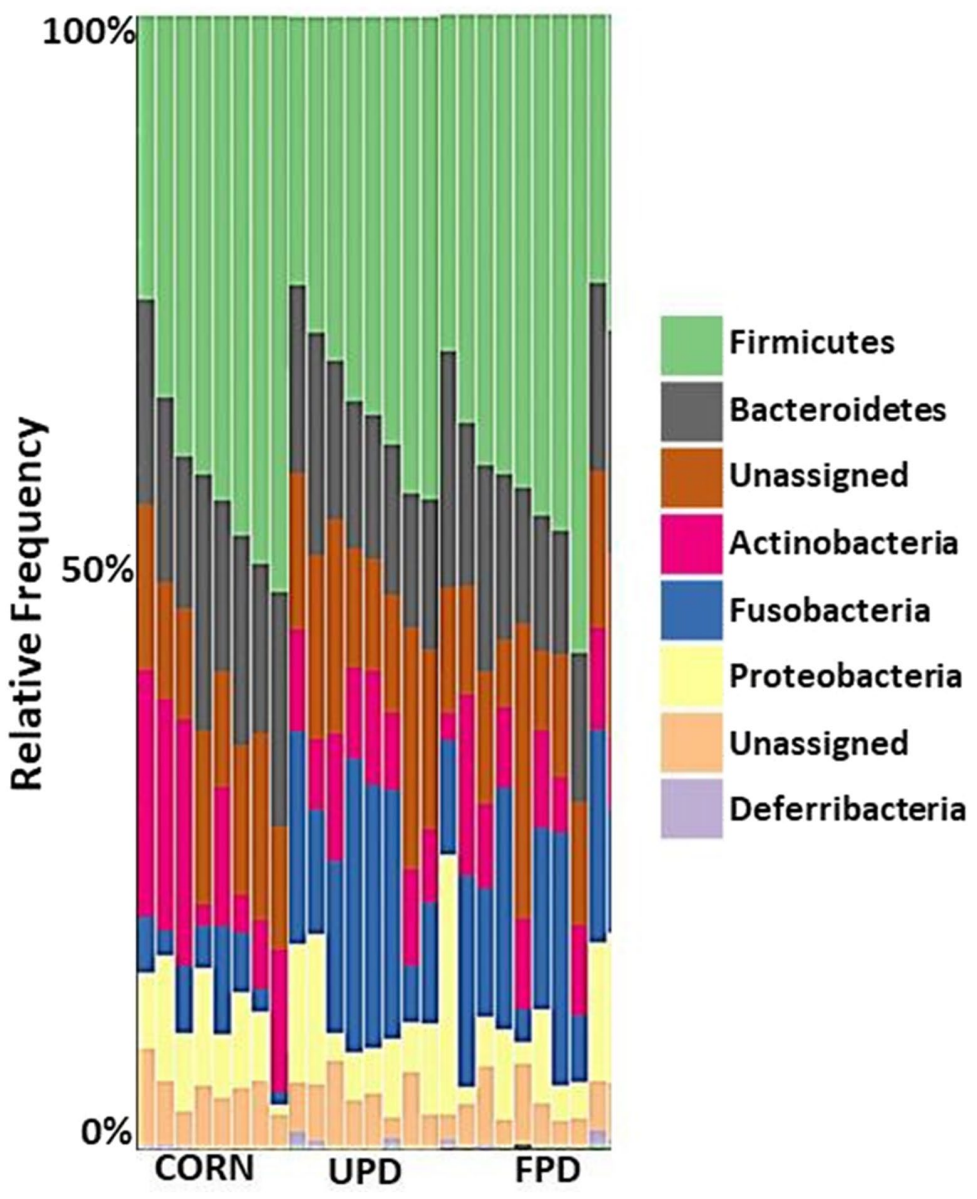
Median percent of Operational Taxonomic Unit (OTU) representing bacterial phylum in the stool of dogs (*n* = 8) fed different diets for 20 days: UPD, unfermented pea diet; FPD, fermented pea diet; corn, control diet. The relative abundances are inferred from 16S rRNA. The X and Y axes represent the sample name and percentages of bacterial taxa, respectively.

**Figure 6:**
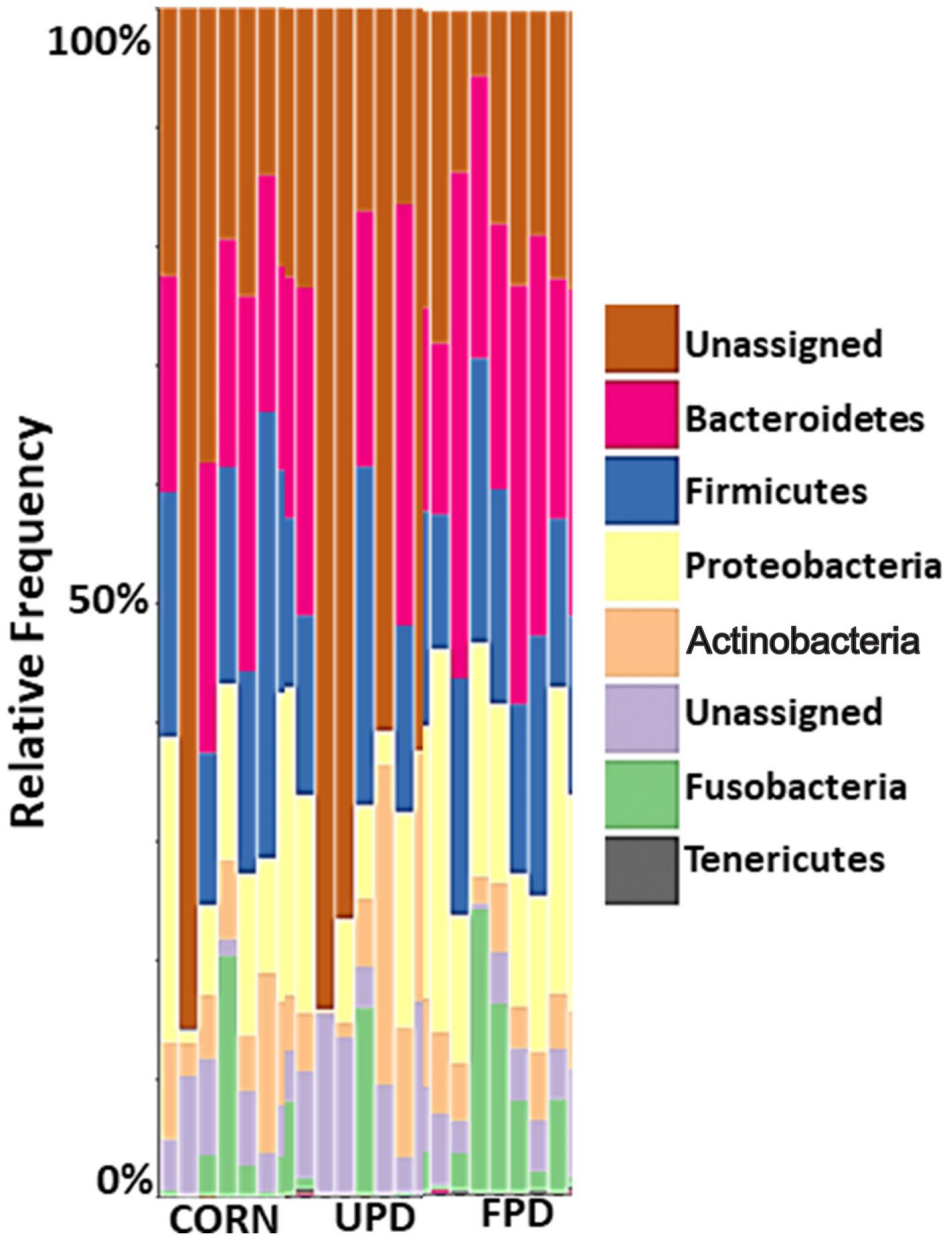
Median percent counts of Operational Taxonomic Units (OTU) representing bacterial phylum in the stool of cats (*n* = 7) fed different diets for 20 days: UPD, unfermented pea diet; FPD, fermented pea diet; corn, control diet. The relative abundances are inferred from 16S rRNA. The X and Y axes represent the sample name and percentages of bacterial taxa, respectively.

*Firmicutes*, *Bacteroidetes*, *Actinobacteria*, *Fusobacteria*, and *Proteobacteria* were the major bacteria phylum found in the feces of dogs and cats (Figures 5, 6, respectively). Dogs showed an increased load of *Fusobacteria* when fed both pea diets compared to the corn diet (Figure 5). Similarly, cats showed increased intestinal *Fusobacteria*, but only when fed the fermented pea diet (Figure 6). No apparent differences in relative frequency were observed in fecal bacterial composition between the corn and unfermented pea diets in cats (Figure 6).

**Figure 7:**
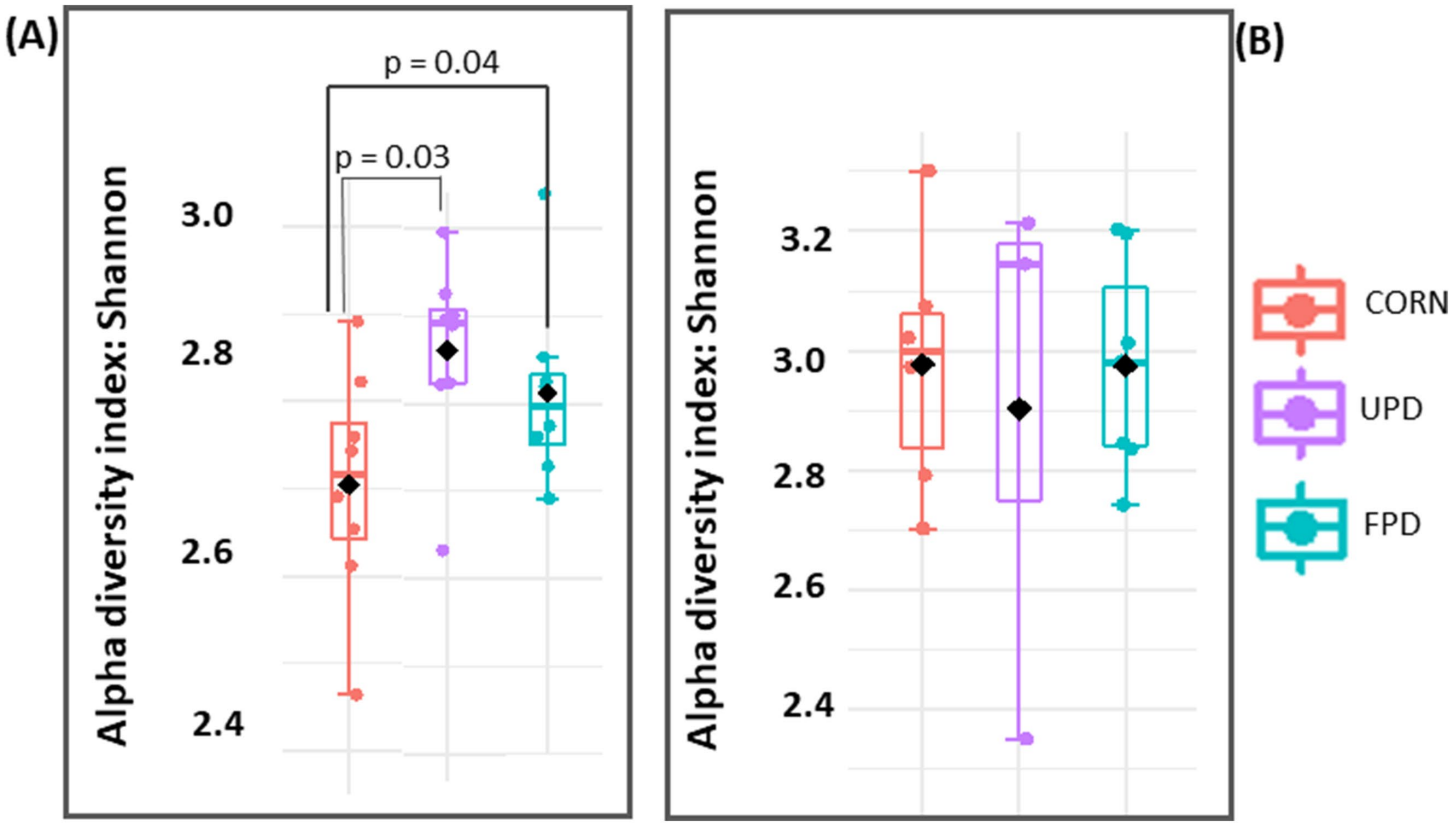
Alpha diversity of intestinal microbiome after 20 days of feeding test diets: Shannon Index based on Operational Taxonomic Unit (OTU) abundance for **(A)** dogs (*n* = 8) and **(B)** cats (*n* = 7) fed UPD, unfermented pea diet and FPD, fermented pea diet. *P* < 0.05, Friedman test followed by Dunn’s multiple comparison. The black diamond represents the mean and the middle line represents the median.

**Figure 8:**
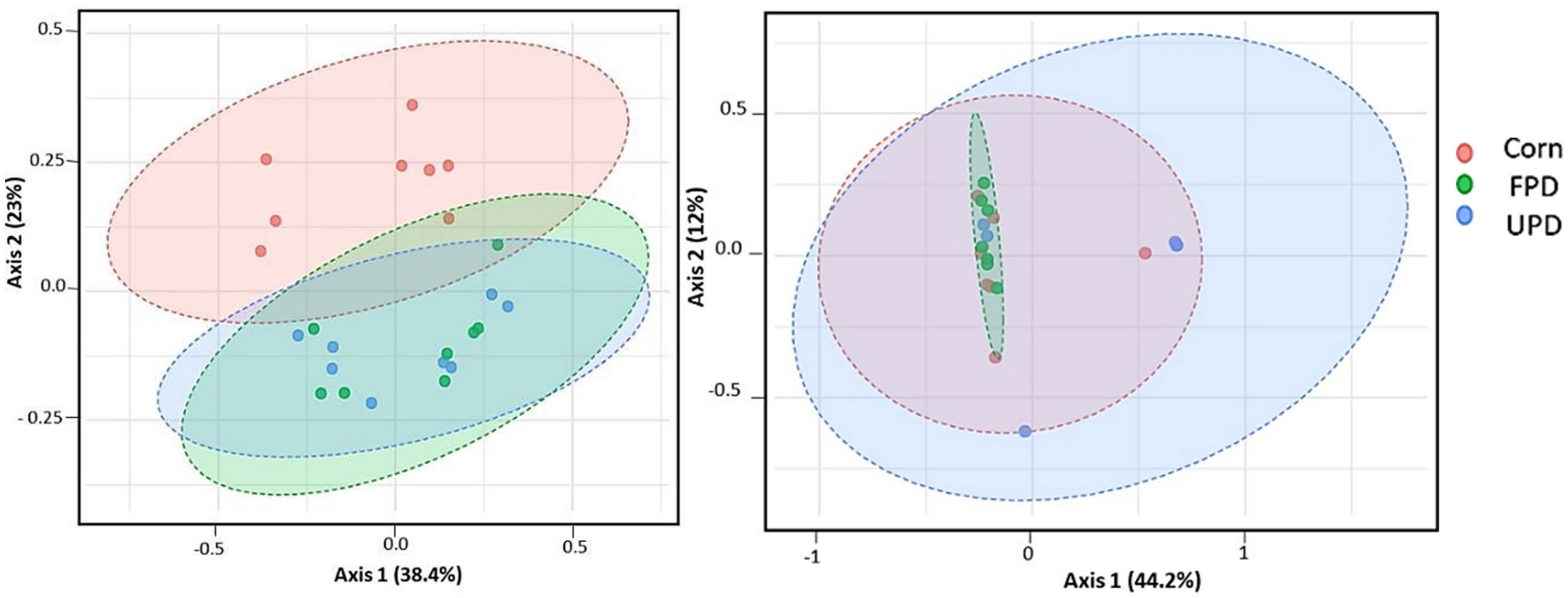
Beta diversity of intestinal microbiome after 20 days of feeding test diets: Principal Components Analysis plot of weighted UniFrac distances for **(A)** dogs (*n* = 8) and **(B)** cats (*n* = 7) fed UPD, unfermented pea diet and FPD, fermented pea diet. A significant (*P* < 0.05, PERMDISP and PERMANOVA) difference among diets was found in dogs but not cats.

Alpha diversity comprising the fecal microbiome increased in dogs fed both pea diets in comparison to corn (Figure 7A). Likewise, beta diversity differed among dogs fed both pea diets in comparison to corn (Figure 8A). In contrast, in cats, no differences were found in either alpha or beta diversity among any of the diets (Figures 7B, 8B, respectively).

**Figure 9:**
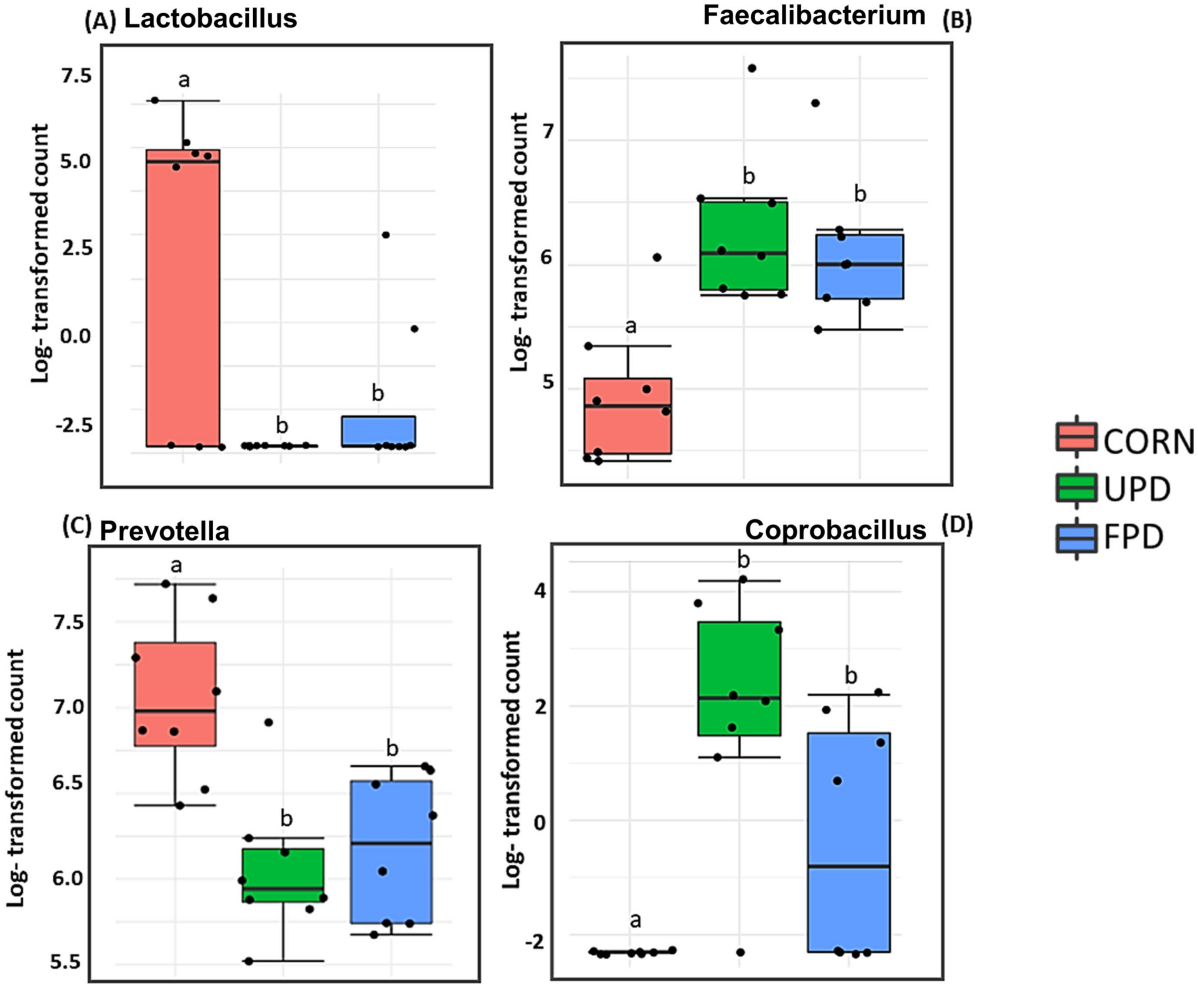
Absolute abundance of bacteria in feces of dogs (*n* = 8) after being fed different diets: UPD, unfermented pea diet; FPD, fermented pea diet and corn for 20 days. **(A)** Lactobacillus, **(B)** Faecalibacterium, **(C)** Prevotella, and **(D)** Coprobacillus. Different letters indicate significant differences after Friedmans test followed by Dunn’s multiple comparison (*p* < 0.05; adjusted FDR).

Differences in taxa abundance of fecal microbiome after 20 days of feeding test diets were only identified in dogs due to the low quality of the samples collected from cats fed the unfermented pea diet (caused diarrhea in cats). Taxa are listed by genus and Figure 9 depicts the ones that presented significant differences in abundance among test diets in dogs. In comparison to the corn diet, *Lactobacillus* and *Prevotella* decreased in dogs fed both pea-based diets (Figure 9). Conversely, *Faecalibacterium* and *Coprobacillus* increased with dogs fed either pea-based diet compared to the corn diet (Figure 9).

## 4 Discussion

Overall, this study revealed that pea-based diets enhance dog and cat metabolic status, indicated by reductions in plasma leptin, inflammation, triglyceride, and cholesterol levels. Furthermore, diet and metabolic status were coincident with changes in intestinal microbial composition and SCFA production in dogs and cats, respectively. Overall, studies correlating microbial composition and metabolic profiles with dietary changes has been underexplored, especially in canine research (39). Despite this, the obese state itself has been linked to similar changes to those observed in the current study for metabolic hormones, lipids, cholesterol and inflammation in dogs after neutering that is coincident with changes in fecal microbiome (40, 41). The, current study was not designed to establish a causal link between the microbial and metabolic changes in companion animals with diet or altered feed intake, but does add some weight to a possible relationship between intestinal microbiome and metabolic health.

### 4.1 Metabolic status and overall health

For body weight and fecal quality, the dogs did not experience any changes after feeding any of the test diets. However, cats experienced weight loss with the unfermented pea diet. This was primarily attributed to either low palatability, discomfort due to diarrhea or both. All the cats refused to consume the unfermented pea diet to some degree and this was accompanied by loose stools. In fact, the feeding periods were revised to a shorter time period (20 days) than originally planned (we had targeted 30 days originally) because of ongoing food refusals in the cats and concern that they might develop hepatic lipidosis from prolonged feed intake reductions. Poor palatability of unfermented pea diets in cats has been observed in a previous study from our lab (2). In this previous study, *Candida utilis* fermentation of the whole pea meal was shown to improve palatability in objective tests, where both acceptability and preference in a choice test were assessed (4). Of interest, the previous study detected similar acceptability between fermented and unfermented pea-based diets based on single, short-term feedings, but a significantly lower preference for the unfermented diet in a choice test. However, the food refusals by cats in the current study instead qualitatively suggest lower acceptability of the unfermented pea diet compared to the other diets, but that the acceptability is latent. Reasons for the delayed decrease in acceptability are unclear, but would be consistent with gastrointestinal upset and abdominal discomfort that often accompany diarrhea. The current study used a more purified pea product, wet processed pea starch that is >95% starch than our previous study (4) which one might hope would remove factors that reduce palatability of peas. However, results of this study clearly show the unfermented pea starch still had palatability issues despite higher starch purity. Moreover, the current study confirms our previous findings that fermentation with *Candida utilis* improves palatability for cats since they consumed the fermented pea diet without experiencing any weight loss. Moreover, fermentation resolved the diarrhea issue in cats we had observed after feeding the unfermented pea diet.

Previous studies that demonstrated poor metabolic status is associated with higher leptin and decreased adiponectin concentrations in companion animals (19, 42–45). Excluding results in the current study with cats fed unfermented peas that led to food refusals and diarrhea, beneficial body weight changes were not detected. Dogs and cats were maintained at ideal body condition in the current study and thus weight changes were not desirable. Despite a lack of weight change, both species showed triglyceride and cholesterol decreases associated with improved metabolic health that coincided with beneficial decreases in leptin, but no adiponectin increase after eating the pea-based diets. Reduced food intake by itself has been reported to cause reductions in leptin levels in obese cats (46, 47) which agrees with the observed leptin decrease in cats fed unfermented peas that had decreased food intake in the current study. In contrast, the same cannot be said for the leptin decrease in dogs fed either pea-based diet, nor in cats for the fermented pea diet since food intake was not altered in these groups. This demonstrates metabolic benefits of pea-based diets independent of weight change or food intake in the current study.

Decreased adiponectin and increased leptin are associated not only with weight gain and obesity, but also with increased chronic inflammation in dogs and cats (33, 48). In dogs in the current study, reduced white blood cell, lymphocyte and neutrophil counts suggests inflammation was reduced after consuming the pea diets. These findings agree with a previous study reporting decreased C-reactive peptide, interleukin-6 and leptin in dogs fed a high protein, high corn-fiber diet (20). However, two additional markers of inflammation examined in the current study, NLR and PLR provided conflicting results in dogs. While NLR did not change with any of the diets tested in either species, canine PLR increased with both pea diets. An increased PLR could be interpreted as an increased, not decreased inflammation. However, in this case, the dog platelet numbers did not change with diet, so the increased PLR change may be secondary to the decrease in lymphocyte numbers and instead should be interpreted as an overall decrease in inflammation. In cats, the reduced PLR observed after consuming the fermented pea diet in the current study was similarly not due to a change in platelet count. Instead, the reduced PLR seems to be due to a non-significant trend to increased lymphocyte number, accompanied by no significant changes in any white blood cell numbers. Thus, it appears cats did not show any improvement in inflammatory status with the diets tested in the current study. Taken together, NLR and PLR parameters have been explored for dog oncology but show high variability among breeds and interpretation of these ratios should be taken with caution (49). Pea diets, particularly when fermented show potential to reduce canine inflammation, but whether this is common to all fiber-rich diets or not is unclear and should be investigated further.

Despite some differences found in blood biochemistry of dogs fed different diets, most values were still within the reference range and changes were not considered clinically significant. In cats after feeding the unfermented pea diet, blood chemistry changes included lower serum total protein and globulin compared to the other two test diets. This closely aligns with food refusals and diarrhea observed with the unfermented pea diet since reduced protein intake will directly influence serum protein levels (50). In contrast, both pea diets (fermented and unfermented) caused other changes in cat blood chemistry, including higher sodium, bicarbonate, creatinine and total bilirubin, but lower anion gap. While still within clinical normal limits and likely not of concern, this pattern of change is not readily explained.

Both pea-based diets resulted in additional positive metabolic changes for both dogs and cats. Specifically, lower plasma cholesterol levels were observed for both species after consuming both the unfermented pea diet compared to the fermented pea and corn diets. It has been established that the consumption of peas results in reduced cholesterol and triglyceride levels in humans (51), while consuming a high protein, high corn fiber diet has been shown to have similar effects in dogs (20, 21). This is generally attributed to soluble fiber, common to both pea starch and corn fiber, increasing viscosity of the intestinal digesta, thereby trapping cholesterol and lipid and preventing their absorption (51, 52). In the current study, the fermented pea diet resulted in an intermediate cholesterol level between the unfermented pea diet and the highest value observed after feeding the corn diet in dogs. If anti-nutritional factors could be an additional contributor to cholesterol and triglyceride lowering effects, then fermentation may degrade some of these compounds and you would expect a difference between the pea diets. In support of this hypothesis, we measured tannins and phenols in the fermented versus the unfermented pea flours using the Folin–Ciocalteu method in the same cohort of dogs and confirmed that levels of these anti-nutritional factors did decrease ~10-fold after fermentation (4). Peas are high in all types of fiber, including soluble fibers (51, 52), but all three test diets including the corn diet had added pea fiber. Unlike pea fiber, pea starch is more likely to have retained low molecular weight soluble fiber that would contribute to viscosity of the intestinal digesta (53). Taken together with previous studies reporting similar effects with corn fiber in dogs (20, 21), this suggests soluble fiber and viscosity may instead have played a bigger role in decreasing triglycerides in both species and decreasing cholesterol in cats since fermentation did not change these beneficial effects. Overall, these results support the hypothesis that pea-based diets improve cholesterol and lipid handling, further contributing to improved metabolic health in dogs and cats.

### 4.2 Intestinal health

Evaluating cat intestinal health revealed a surprising find with SCFA content between the corn and both pea-based diets. Specifically, cats had higher fecal butyrate when fed the corn diet in comparison to both pea-based diets. This is an interesting find since previous companion animal research indicates that increased intestinal butyrate concentrations are linked to consumption of feed containing high fiber and other prebiotics (32, 54, 55), similar to the fermented pea diet in the current study. However, examining the test diet formulations closer, we found that the corn diet in the current study had a higher inclusion of pea fiber compared with both pea-based diets. We did not measure fiber fractions in our test diet in the current study. However, if the fiber type found in pea fiber is fermentable and could drive microbial fermentation; this would explain the higher fecal butyrate concentrations in the cats after eating the corn-based diet. In contrast, the lack of change in butyrate in dogs with the same diet argues against this or at least suggests that species differences in microbiome elicit differences in fermentation. In comparison, the pea-based diets had less pea fiber and instead used highly purified pea starch that would be enriched in resistant starch and low molecular weight fiber compared to the corn diets in the current study. On the other hand, research has also reported that obese individuals can have impaired SCFA absorption, resulting in higher fecal SCFA levels in humans and dogs (56), while knowledge in cats is more limited. The mechanisms behind the observed higher fecal SCFA concentration in cats fed the corn diet in the current study is unknown and should be further investigated.

In the current study in cats, the predominant two bacterial phyla based were *Bacteroides* and *Firmicutes*, agreeing with a previous study (57). The abundance of these two phyla remained qualitatively similar for all diets tested. When evaluating the cat fecal microbial alpha and beta diversity in the current study, diet had no effect. While age has been reported to change intestinal microbiota in cats (58), no previous studies have examined dietary effects on cat fecal microbiome. Previous studies suggested that the high inter-individual variability in domestic cats makes it difficult to obtain concrete results about their intestinal microbiome (59, 60). Moreover, the decreased feed intake with the unfermented pea diet resulted in reduced stool production that was of a poor quality for collection, leading to a low yield of genetic material obtained from the fecal samples and low numbers of reads for the DNA sequencing analyses in cats. Overall, more research is needed in improving the techniques used to evaluate any possible differences in the cat intestinal microbiome with diet.

In the current study in dogs, the predominant two bacterial phyla were similar to cats: *Firmicutes* and *Bacteroides*. The finding that *Bacteroides* predominates in dog feces agrees with previous studies using dog samples (17, 73). However, the second most abundant phyla in canine feces differs in previous studies, with reports of either *Firmicutes* (73) or *Fusobacteria* (17). Differences in findings may be due to diet since resistant starch from high amylose corn or potato starch were tested in these other studies, respectively. In contrast to cats, dog fecal microbiome alpha diversity increased with both pea-based diets. Moreover, beta diversity differed among dogs fed corn or pea-based diets. Specifically, *Lactobacillus* was depleted in feces from dogs fed peas instead of corn. While no deleterious health effects were observed in the current study in dogs with lower Lactobacilli, many previous studies report that Lactobacilli are positively linked to intestinal health in different species including dogs (55, 61, 62). A possible explanation to the higher population of *Lactobacillus* in the intestine of dogs fed corn may be due to the higher inclusion of pea fiber. In fact, studies have previously shown that the consumption of diets rich in fiber positively correlates with increased intestinal levels of *Lactobacillus* in dogs, cats, and humans (55, 61, 62). Even though levels of *Lactobacillus* in dogs fed both pea diets were lower than corn, dogs fed the fermented pea diet showed a modest improvement in the population of *Lactobacillus* compared to those fed unfermented pea diet. This might be related to the prebiotic compounds present in the *C. utilis*-cell wall (6, 14, 63).

Similarly, the intestinal *Faecalibacterium* population was found to increase with the consumption of pea diets in dogs, agreeing with another study reporting similar increases in dogs fed higher dietary potato fiber (17). Although causal links have not been established, lower intestinal *Faecalibacterium* is associated with obesity and diabetes in humans, dogs and cats as well as Crohn’s disease in humans (17, 18, 64–66). Additionally, dogs suffering from inflammatory bowel disease have lower amounts of *Faecalibacterium* DNA (18, 64, 66). Therefore, the higher concentration of fecal *Faecalobacterium* in dogs fed peas further suggests a potentially beneficial effect of pea-based diets in improving gut health.

Finally, we identified conflicting results regarding *Prevotella* and *Coprobacillus*. Fecal *Prevotella* was reduced in dogs fed the pea-based diet in comparison to the corn diet. The role of *Prevotella* in intestinal health is not yet fully understood, with conflicting research suggesting increased populations can improve glucose handling but has also been linked to intestinal inflammatory disease in humans (67, 68). Additionally, the *Prevotella* genus includes many species, requiring further studies needed to understand the role of specific species in the dog gut (67, 68). Similarly, levels of intestinal *Coprobacillus* was lower in the fermented pea-based diet which can possibly be explained with the supplementation of the yeast cell wall based on studies in humans and cats (69, 70). However, the significance of this result is not fully understood. Overall, the general trend for changes in abundance of specific bacteria seems to be following a beneficial impact on intestinal health for dogs fed pea-based diets in comparison to the corn diet, with only minor additional effect when pea starch was fermented.

### 4.3 Limitations

A major limitation to the current study is the small sample size (*N* = 8 beagles, *N* = 7 cats). Unfortunately, one of the cats developed irritable bowel syndrome (IBD) and reduced the sample size to seven for cats. However, the small sample size was counteracted by using a randomized crossover design and repeated measures statistical analyses. Another major limitation was that we did not include a test diet where *C. utilis* yeast extracts alone were added to an unfermented pea diet. Several previous studies using a *Saccharomyces cerevisiae* fermentation product as a supplement in dog food have reported anti-inflammatory effects and improved intestinal health (8, 9). Thus, we cannot say whether dietary inclusion of yeast fermentation products produced the additional health benefits observed in this study or whether fermentation changed an important (anti-)nutritional factor in peas that then produced health benefits. In support of the latter possibility, a previous study from our group showed that fermentation decreased crude fiber and resistant starch, but increased slowly digestible starch fractions (4). Moreover, while most proximate analyses remained unchanged between fermented and unfermented pea starch, fermentation was noted to decrease phenolic content and possibly other anti-nutritional factors in pea flour. Therefore, future studies should examine whether simple inclusion of yeast fermentation products in pea-based diets can recapitulate all observed health benefits in dogs. Another limitation would have been to verify that the blood parameters returned to baseline values before and after the wash-out commercial diets to ensure the washout periods served their purpose. Additionally, accounting for confounding variables such as weight loss and food refusals, especially with the cats, was a major limitation since it would have been difficult to statistically distinguish. These variables should be addressed in future studies.

## 5 Conclusion

In summary, the current study confirmed results of many other previous studies that intestinal microbial composition in dogs is influenced by diet. In contrast, we can only say that cat fecal SCFA was instead altered by diet because fecal samples were of insufficient quality and consistency to produce reliable microbiome results. Additionally, the *C. utilis*-fermented pea starch diet improved dog fecal microbial diversity, while both pea diets increased richness and evenness in the microbial population, all indications of improved gut health. These microbiome and fecal SCFA changes were associated with decreases in plasma cholesterol, triglycerides and leptin, indicating improved metabolic health in both dogs and cats fed pea-based diets. Taken together, the use of yeast-fermented pea starch should be further explored as a novel functional ingredient in the pet food industry.

## Data Availability

The datasets presented in this study can be found in the GenBank repository (BioProject accession PRJNA1266192). All other data can be found in the article/Supplementary material.
