# Supplementary material for: Effects of feeding Candida utilis-fermented pea starch on overall, metabolic and intestinal health of dogs and cats
Source: Front Vet Sci. 2025 Jun 9;12:1542484. doi: 10.3389/fvets.2025.1542484 (PMC12183961; doi:10.3389/fvets.2025.1542484)
Supplement: Supplementary file 1 [file Data_Sheet_1.docx]

Supplementary Material

**Table S1**. Dog blood cell count and biochemistry profile after fed test diets for 20 days

| **Blood parameters** | **Reference range** | **Corn diet** | **UPD** | **FPD** |
| --- | --- | --- | --- | --- |
| Sodium (mmol/l) | 140 - 153 | 147 ± 0.4 | 147 ± 0.3 | 148 ± 0.2 |
| Potassium (mmol/L) | 3.8 - 5.6 | 4.6 ± 0.06 | 4.6 ± 0.05 | 4.6 ± 0.06 |
| Chloride (mmol/L) | 105-120 | 112 ± 0.4 | 111± 0.4 | 111 ± 0.4 |
| Bicarbonate (mmol/L) | 15 -25 | 20 ± 0.7 | 20 ± 0.6 | 22 ± 0.5 |
| Anion gap (mmol/L) | 12-26 | 19 ± 0.6 | 20 ± 0.8 | 19 ± 0.4 |
| Calcium (mmol/L) | 1.91-3.03 | 2.4 ± 0.02 | 2.5 ± 0.02 | 2.5 ± 0.02 |
| Phosphorus (mmol/L) | 0.63-2.41 | 1.2 ± 0.05 | 1.1 ± 0.05 | 1.2 ± 0.04 |
| Magnesium (mmol/L) | 0.70-1.16 | 0.8 ± 0.01 | 0.7 ± 0.01 | 0.8 ± 0.01 |
| Urea (mmol/L) | 3.5-11.4 | 5.3 ± 0.2 | 5.2 ± 0.2 | 5.5 ± 0.4 |
| Creatinine (mmol/L) | 41-121 | 65 ± 1 | 62 ± 2 | 60 ± 5 |
| Amylase (U/L) | 343-1375 | 523 ± 24ª | 516 ± 27ª | 468 ± 17ᵇ |
| Lipase (U/L) | 25-353 | 56 ± 7 | 52 ± 8 | 43 ± 5 |
| Glucose (mmol/L) | 3.1-6.3 | 4.8 ± 0.10 | 4.9 ± 0.10 | 4.8 ± 0.08 |
| Cholesterol (mmol/L) | 2.7- 5.94 | 4.5 ± 0.2ª | 4.1 ± 0.2ᵇ | 4.4 ± 0.2ª |
| Total bilirubin (μmol/l) | 1.0-4.0 | 1.3 ± 0.3 | 1.4 ± 0.1 | 1.7 ±0.2 |
| Direct bilirubin (μmol/l) | 0.0- 2.0 | 0.3 ± 0.10 | 0.6 ± 0.06 | 0.6 ± 0.09 |
| Indirect bilirubin (μmol/l) | 0.0- 2.5 | 1 ± 0.1 | 0.8 ± 0.1 | 1 ± 0.1 |
| ALK phosphatase (U/L) | 9-90 | 29 ± 5 | 25 ± 3 | 25 ± 3 |
| GGT (U/L) | 0-8 | 0.8 ± 0.4ª | 2.3 ± 0.4ᵇ | 2.7 ± 0.3ᵇ |
| ALT (U/L) | 19-59 | 27 ± 4 | 23 ± 1 | 24 ± 2 |
| CK (U/L) | 51-418 | 188 ± 25 | 180 ± 15 | 182 ± 16 |
| Total protein (g/L) | 55-71 | 56 ± 0.8 | 56 ± 0.8 | 56 ± 0.7 |
| Albumin (g/L) | 32-42 | 35 ± 0.6 | 36 ± 0.5 | 36 ± 0.6 |
| Globulin (g/L) | 20-34 | 20 ± 0.5 | 19 ± 0.5 | 20 ± 0.3 |
| A:G ratio | 1.06-1.82 | 1.7 ± 0.05 | 1.8 ± 0.04 | 1.7 ± 0.04 |
| WBC (x10⁹/L) | 4.9-15.4 | 6.0 ± 0.3ª | 4.7± 0.2ᵇ | 4.7 ± 0.3ᵇ |
| RBC (x10¹²/L) | 5.8-8.5 | 6.0 ± 0.2 | 6.0 ± 0.1 | 6.0 ± 0.1 |

Data is mean ± SEM, n=8. One-way repeated measures ANOVA with post hoc LSD. Values in a row with superscripts without a common letter are significantly different (P < 0.05). UPD = unfermented pea diet; FPD = fermented pea diet; ALK= alkaline phosphatase, GGT= gama-glutamyl transferase, ALT= alanine transaminase, CK= creatine kinase, A:G ratio= albumin/globulin ratio, WBC= white blood cells, RBC= red blood cells

**Table S2**. Cat blood cell count and biochemistry profile after fed test diets for 20 days

| **Blood parameters** | **Reference range** | **Corn diet** | **UPD** | **FPD** |
| --- | --- | --- | --- | --- |
| Sodium (mmol/l) | 147-160 | 152 ± 0.6ᵃ | 154 ± 0.6ᵇ | 153 ± 0.4ᵇ |
| Potassium (mmol/L) | 3.9-5.5 | 4.7 ± 0.05 | 4.7 ± 0.09 | 4.5 ± 0.10 |
| Chloride (mmol/L) | 111-125 | 116 ± 0.9ᵃ | 118 ± 0.7ᵇ | 116 ± 0.4ᵃᵇ |
| Bicarbonate (mmol/L) | 11 -22 | 14 ± 0.8ᵃ | 17 ± 0.4ᵇ | 17 ± 0.2ᵇ |
| Anion gap (mmol/L) | 15-30 | 27 ± 0.9ᵃ | 23 ± 0.4ᵇ | 23 ± 0.5ᵇ |
| Calcium (mmol/L) | 2.26-2.86 | 2.4 ± 0.02 | 2.4 ± 0.02 | 2.5 ± 0.03 |
| Phosphorus (mmol/L) | 1.08-2.21 | 1.5 ± 0.06 | 1.4 ± 0.04 | 1.4 ± 0.05 |
| Magnesium (mmol/L) | 0.74-1.12 | 0.9 ± 0.01ᵃ | 0.9 ± 0.01ᵃᵇ | 0.8 ± 0.01ᵇ |
| Urea (mmol/L) | 6-11.4 | 7.6 ± 0.3 ᵃ | 6.8 ± 0.4 ᵇ | 7.2 ± 0.3 ᵃᵇ |
| Creatinine (mmol/L) | 78-178 | 118 ± 2.8ᵃ | 149 ± 3.4ᵇ | 153 ± 2.3ᵇ |
| Glucose (mmol/L) | 3.5-8.1 | 3.9 ± 0.2ᵃ | 4.3 ± 0.2ᵃᵇ | 4.5 ± 0.2ᵇ |
| Cholesterol (mmol/L) | 1.62-4.32 | 7.1 ± 0.5ᵃ | 4.5 ± 0.4ᵇ | 5.0 ± 0.5^c^ |
| Total bilirubin (μmol/l) | 0.0-3.0 | 0.3 ± 0.06ᵃ | 0.7 ± 0.07ᵇ | 0.7 ± 0.10ᵇ |
| GGT (U/L) | 0-6 | 0 | 0 | 0 |
| ALT (U/L) | 22-90 | 64 ± 8 | 59 ± 7 | 52 ± 4 |
| GLDH (U/L) | 1-5 | 2.7 ± 0.4 | 4.2 ± 0.4 | 3.1 ± 0.8 |
| CK (U/L) | 75-471 | 281 ± 59ᵃᵇ | 151 ± 16ᵃ | 125 ± 16ᵇ |
| Total protein (g/L) | 53-84 | 77 ± 2ᵃ | 69 ± 3ᵇ | 76 ± 2ᵃ |
| Albumin (g/L) | 28-43 | 35 ± 0.6ᵃᵇ | 35 ± 1.0ᵃ | 38 ± 0.9ᵇ |
| Globulin (g/L) | 23-45 | 41 ± 1ᵃ | 34 ± 2ᵇ | 38 ± 1ᵃ |
| A:G ratio | 0.77-1.64 | 0.8 ± 0.03ᵃ | 1 ± 0.05ᵇ | 1 ± 0.03ᵇ |
| WBC (x10⁹/L) | 4.2-13 | 10 ± 0.8 | 11 ± 0.8 | 12 ± 1.0 |
| RBC (x10¹²/L) | 6.2-10.6 | 10 ± 0.4 | 10 ± 0.5 | 10 ± 0.5 |

Data is mean ± SEM, n=7. One-way repeated measures ANOVA with post hoc LSD. Values in a row with superscripts without a common letter are significantly different (P < 0.05). UPD = unfermented pea diet; FPD = fermented pea diet; GGT= gama-glutamyl transferase, ALT= alanine transaminase, GLDH= glutamate dehydrogenase, CK= creatine kinase, A:G ratio= albumin/globulin ratio, WBC= white blood cells,
